# Supplementary figures and images for: Fungi and bacteria occupy distinct spatial niches within carious dentin
Source: PLoS Pathog. 2024 May 28;20(5):e1011865. doi: 10.1371/journal.ppat.1011865 (PMC11161102; doi:10.1371/journal.ppat.1011865)

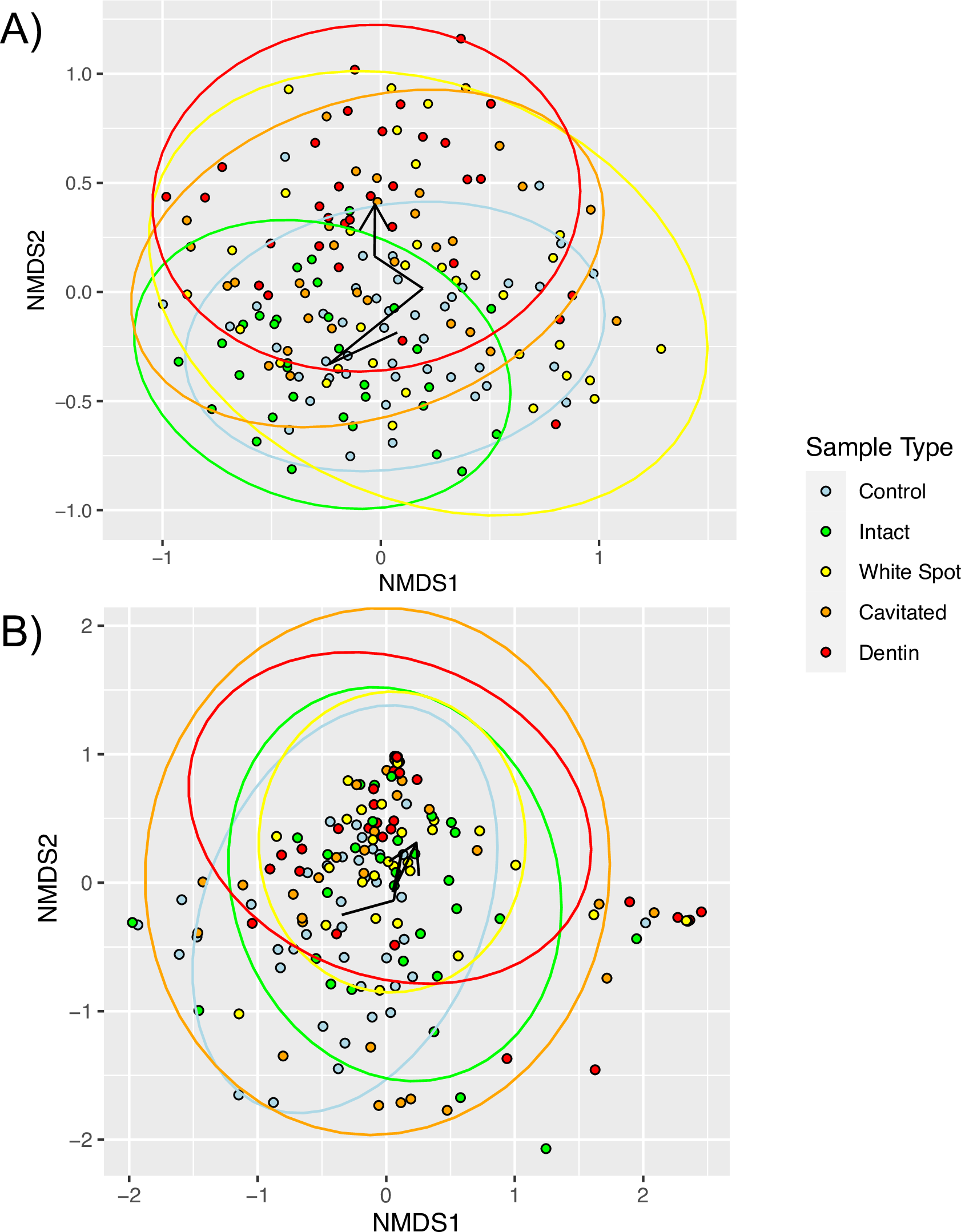

Supplement: S1 Fig — The black arrows show the position of centroids of the various groups and the ellipses show 95% confidence intervals of the centroind position. (A) Bacterial samples (B) Fungal Samples. (TIF) [file ppat.1011865.s001.tif]

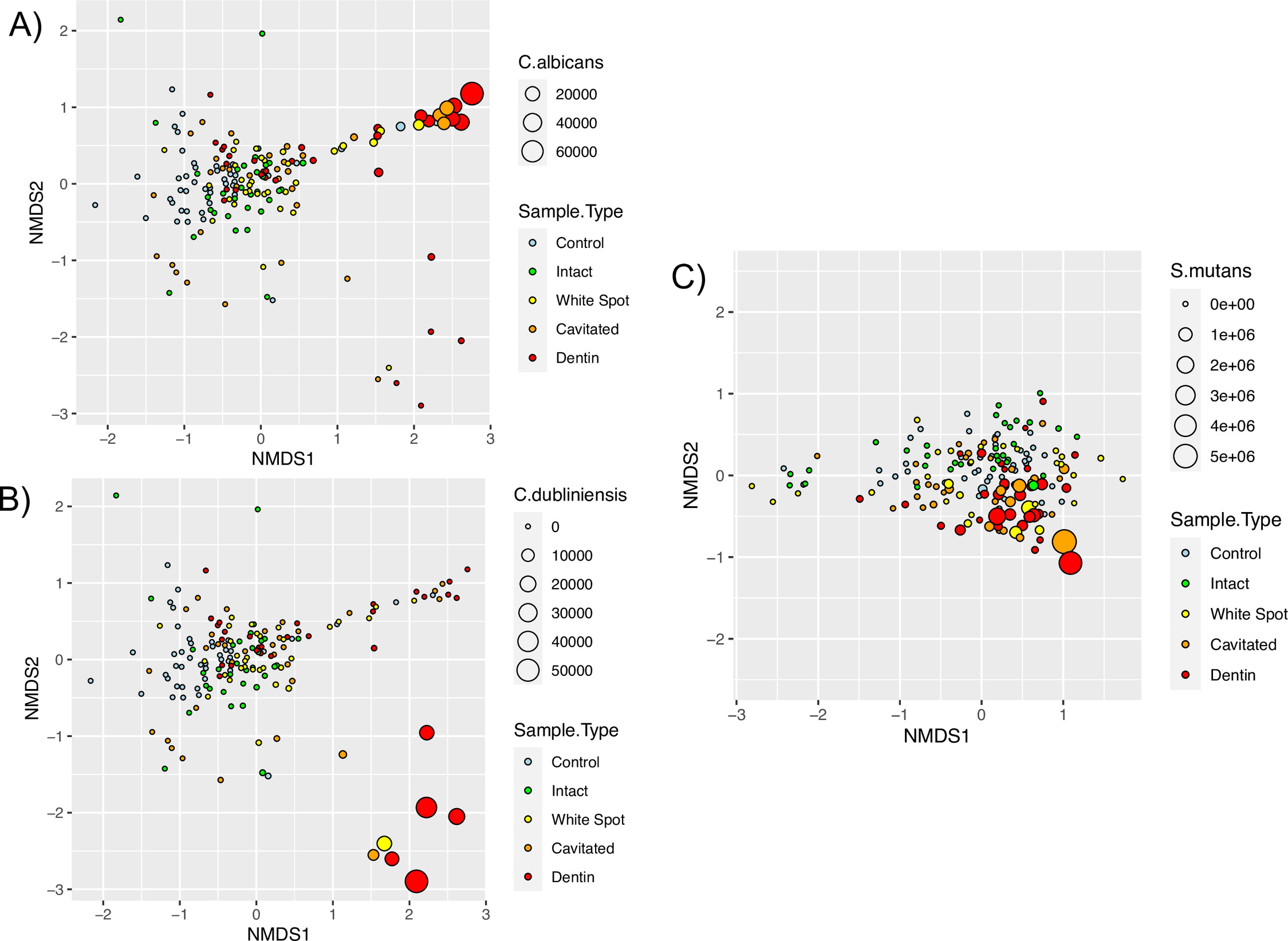

Supplement: S2 Fig — (A) Fungal NMDS sized by Candida albicans abundance (B) Fungal NMDS sized by Candida dubliniensis abundance (C) Bacterial NMDS sized by Streptococcus mutans abundance. (TIF) [file ppat.1011865.s002.tif]

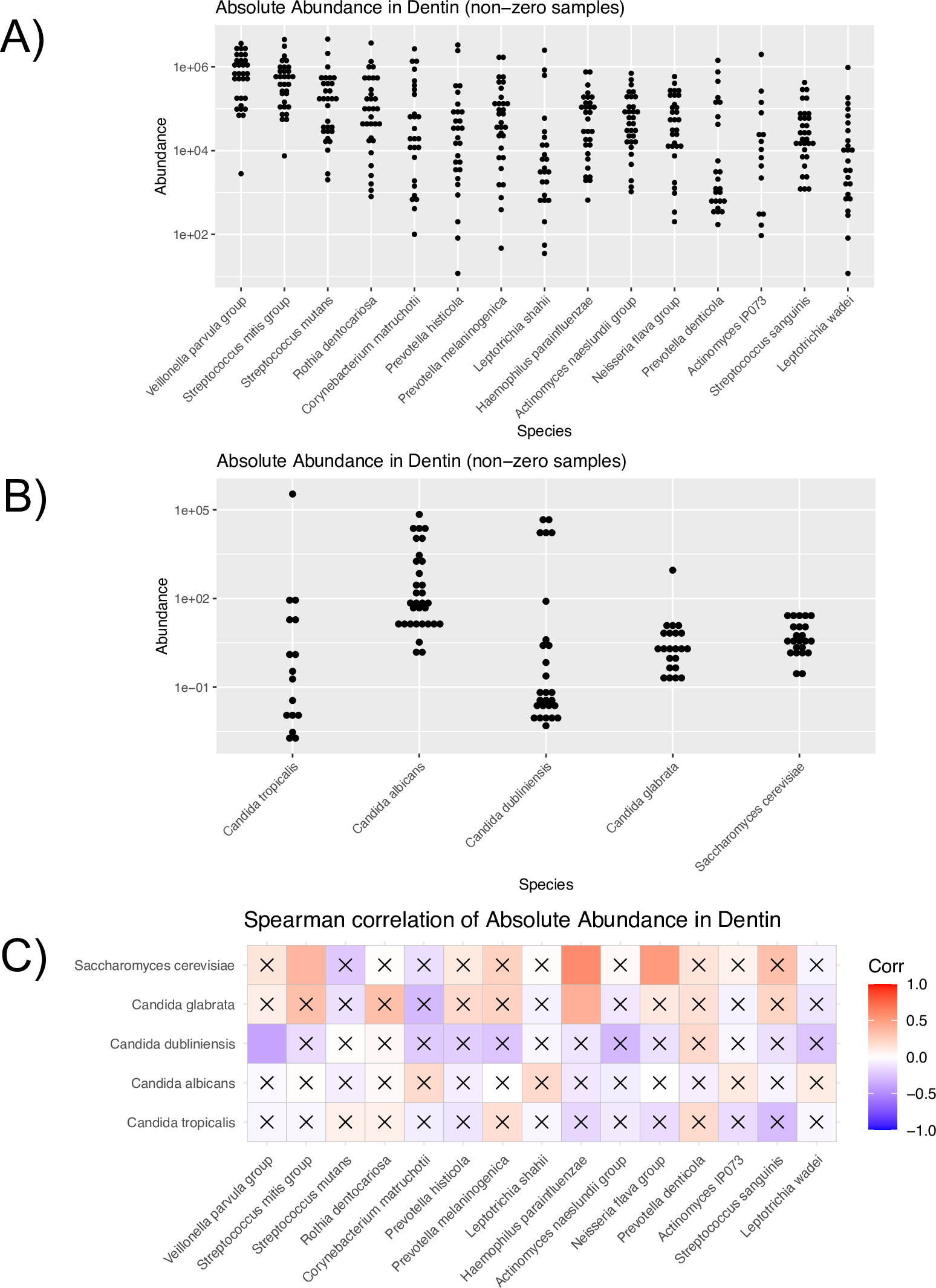

Supplement: S3 Fig — (A) Dotplot of the estimated absolute abundance of 15 bacterial species with the highest mean abundance across dentin lesion samples. (B) Dot plot of the estimated absolute abundance of the 5 fungal species with highest mean abundance across dentin samples. (C) Spearman correlations between the bacterial and fungal groups. Color indicates the strength of correlation and the x’s indicate correlations that are not significantly different than random. (TIF) [file ppat.1011865.s003.tif]

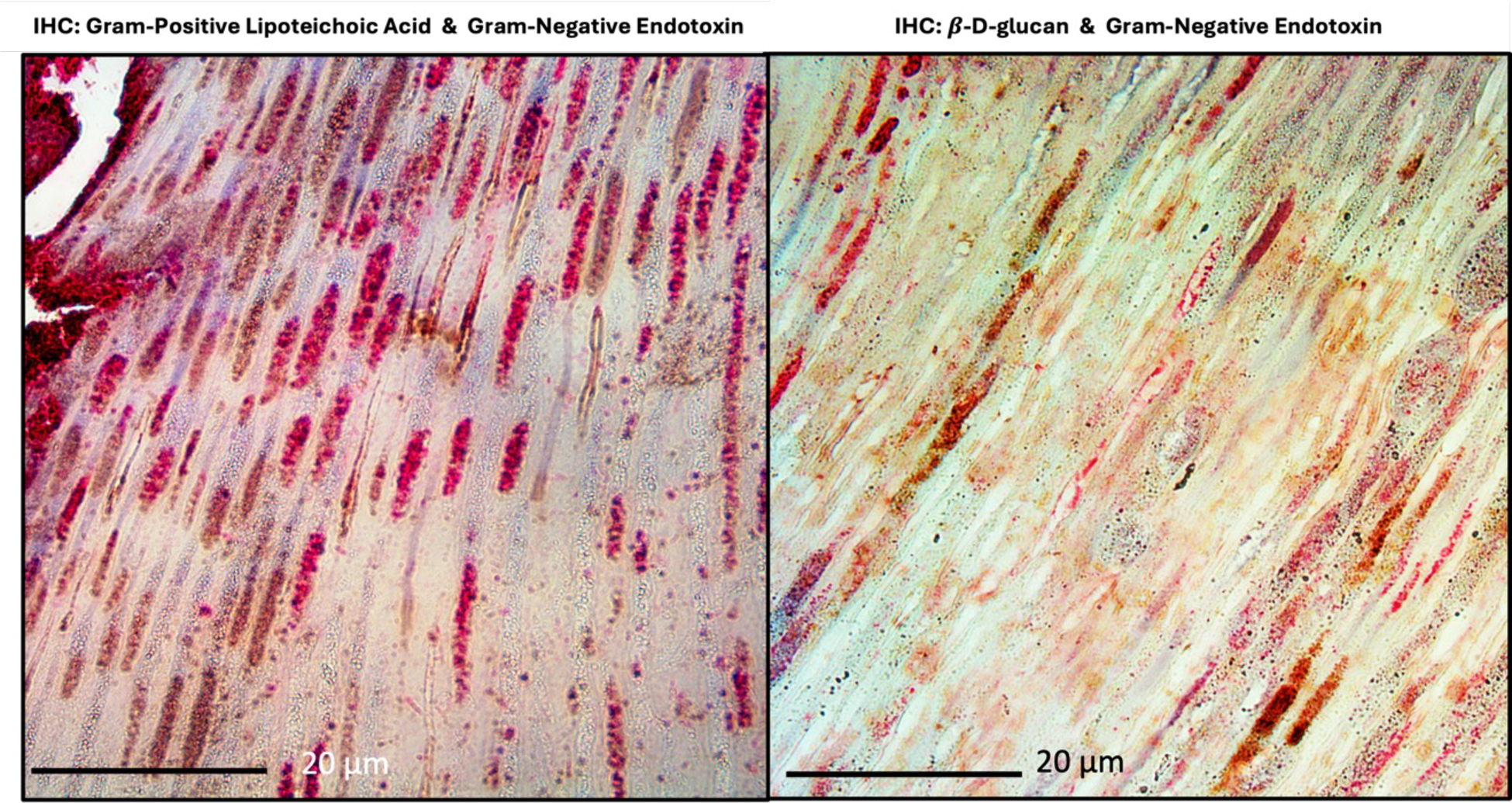

Supplement: S4 Fig — Carious primary teeth were analyzed by IHC to visualize fungi and bacteria. (A) IHC with gram-negative endotoxin (DAB, brown) and lipoteichoic acid (AP, magenta) of carious dentin at 40X magnification. (B) IHC with β-D-glucan (AP, magenta) and Cgram-negative endotoxin (DAB, brown) at 40X magnification. Scale bars are indicated in each panel. (TIF) [file ppat.1011865.s004.tif]
