# Supplementary material for: Fungi and bacteria occupy distinct spatial niches within carious dentin
Source: PLoS Pathog. 2024 May 28;20(5):e1011865. doi: 10.1371/journal.ppat.1011865 (PMC11161102; doi:10.1371/journal.ppat.1011865)
Supplement: S1 Table — R2 values are in the upper right and p values in the lower left. Based on Bray-Curtis dissimilarity of estimated absolute abundance of species incorporating qPCR data. (DOCX) [file ppat.1011865.s005.docx]

| **Bacterial communities** | | | | | |
| --- | --- | --- | --- | --- | --- |
| Plaque type: | Caries-free | Intact enamel | White spot | Cavitated | Dentin |
| Caries-free |  | 0.034 | 0.025 | 0.039 | 0.082 |
| Intact enamel | 0.004 ** |  | 0.037 | 0.045 | 0.082 |
| White spot | 0.022 * | 0.001 *** |  | 0.030 | 0.051 |
| Cavitated | 0.005 ** | 0.001 *** | 0.015 * |  | 0.037 |
| Dentin | 0.001 *** | 0.001 *** | 0.001 *** | 0.003 ** |  |

| **Fungal communities** | | | | | |
| --- | --- | --- | --- | --- | --- |
| Plaque type: | Caries-free | Intact enamel | White spot | Cavitated | Dentin |
| Caries-free |  | 0.063 | 0.025 | 0.039 | 0.082 |
| Intact enamel | 0.001 *** |  | 0.037 | 0.045 | 0.082 |
| White spot | 0.001 *** | 0.126 |  | 0.019 | 0.051 |
| Cavitated | 0.001 *** | 0.010 ** | 0.138 |  | 0.021 |
| Dentin | 0.001 *** | 0.001 *** | 0.001 *** | 0.119 |  |
