# Supplementary material for: Fungi and bacteria occupy distinct spatial niches within carious dentin
Source: PLoS Pathog. 2024 May 28;20(5):e1011865. doi: 10.1371/journal.ppat.1011865 (PMC11161102; doi:10.1371/journal.ppat.1011865)
Supplement: Attachment — Submitted filename: Responses_fungal_dentin.docx [file ppat.1011865.s008.docx]

To: Editorial Staff, PLOS Pathogens

May 8, 2024

I am writing to resubmit our revised manuscript entitled “Fungi and Bacteria Occupy Distinct Spatial Niches within Carious Dentin". We thank the reviewers for their comments and the time taken to review. Below I have included the reviewers’ comments in gray italics and interspersed our responses in black non-italicized. I similarly have appended our responses to the technical issues below that.

Sincerely,

Clifford J. Beall, M.Sc., Ph.D.

Research Associate Professor

Division of Biosciences

The Ohio State University College of Dentistry

**Reviewer Comments and Responses**

***Part I - Summary*** *Please use this section to discuss strengths/weaknesses of study, novelty/significance, general execution and scholarship.*

*Reviewer #1: Overall, this study is an important contribution to our understanding of caries.*

*Reviewer #2: This is an interesting and well-performed study which examines the microbiome and spatial distribution in dentin from Early Childhood Caries (ECC). S. mutans and Veillonella were associated with caries and there was also a higher ratio of fungi to bacteria in more severe lesions. The most notable finding was that fungi and streptococci primarily occupied separate dentinal tubules.*

***Part II – Major Issues: Key Experiments Required for Acceptance*** *Please use this section to detail the key new experiments or modifications of existing experiments that should be absolutely required to validate study conclusions.

Generally, there should be no more than 3 such required experiments or major modifications for a "Major Revision" recommendation. If more than 3 experiments are necessary to validate the study conclusions, then you are encouraged to recommend "Reject".*

*Reviewer #1: Concerns:

1. First, plaque was collected from the surface of exposed dentin. It was not collected from within a tubule. Second, the histology omitted gram-negative bacteria. Consequently, we do not have a complete picture (taxonomic profile) within the tubules and fungi and bacteria may co-colonize tubules more frequently than reported. The environment within a tubule is likely distinct from the surface of dentin. For example, it could be more anaerobic. This would select for different bacteria. This distinction should be made clear. Specifically, that the dentin taxonomic profiles presented do not necessarily reflect the profile within a tubule. The situation is perhaps analogous to the transition between supragingival and subgingival plaque - each harbor distinct communities that contain both gram-positive and gram-negative bacteria.*

We agree that environment within a tubule is likely distinct from the surface of dentin, and that is the major finding we are reporting. The reviewer’s comment made us realize that we incompletely described the sample collection. To clarify, plaque for the microbiome analysis was not collected from the surface of the dentin, but rather carious dentin was collected using a spoon excavator. This detail has been clarified in the manuscript (lines 442-443). This method does break up the dentin and results in sampling of internal tubules.

In response to the comment on gram-negative bacteria, multiplex IHC was performed to identify gram-negative bacteria and either fungi or gram-positive bacteria. The results are now shown in the added Supplemental Figure 4. These images revealed that gram-negative bacteria tend to segregate from fungi and gram-positive bacteria. The text of the Results section was edited to include this result (lines 259-263).

*2. Another issue the small sample size.*

We have put some discussion of sample size in lines 342-346 in the Discussion and fixed incorrect statements in the Methods (lines 430,436). The sample sizes used had been given in the Abstract and we have left those in place. Sample size and power are determined by the variability in the population studied. The sample size of multiple sites from 75 subjects for the microbiome experiments was adequate in that it showed significant differences in microbial community composition and major known pathogens across biologic niches. The data for the histologic investigation showed a high level of consistency across the 20 subjects, so the likelihood that a larger sample size would lead to different conclusions is low.

*Points 1 and 2 are important caveats that need to be addressed and/or clearly stated at least in the abstract.

The distinction between minor and major revision hinges on how the authors wish to interpret and present their findings. For example, a major revision would be the addition of histology for gram-negative bacteria.*

*Reviewer #2: 1. Include a rationale for the use of P. gingivalis DNA as the standard for bacterial quantitation. It seems an unusual choice for a caries study. How does its rDNA gene load compare with that of the oral streptococci and other species that were identified in the study*

In lines 460-470 we have included a rationalization for the use of *P. gingivalis* which is that it has a medium density of rRNA operons and we have used it in previous studies.

*2. Also include the rationale for collecting samples for microbiome or for imaging a two different sites and explain how clinical diagnosis was calibrated. Imminent exfoliation is given as a reason for extraction in both control and ECC patients.*

The International Caries Detection and Assessment System (ICDAS) is a standard used in clinical dentistry to score caries severity. This standard was used at both sampling locations. We have included this detail in lines 437-438. Imminent exfoliation allowed the extraction of human teeth that could not otherwise be ethically obtained.

***Part III – Minor Issues: Editorial and Data Presentation Modifications*** *Please use this section for editorial suggestions as well as relatively minor modifications of existing data that would enhance clarity.*

*Reviewer #1: Minor point
L-278 "A single fungal species was almost always dominant within an individual carious tooth; co-colonizing fungi of distinct species was seldom observed."  For these teeth, were multiple ASVs of the same species observed? Did certain ASVs dominate? It maybe of interest to consider intra-specific variation. Considerable variation in biochemical characteristics among strains of a species may exist.*

We did an analysis of fungal sequences with the DADA2 ASV technique but found multiple C. albicans ASVs even in the positive control which was made from a mix containing a single C. albicans strain. Although we did not have C. dubliniensis DNA in the control we observed that one C. dubliniensis ASV accounts for 99.9% of the total of the experimental samples. We have included these observations in lines 226-233 but overall it appears ASV analysis is not definitive in determining the number of strains for either species.

*Reviewer #2: As S. mutans and C. albicans can be found intermixed and co-adhered to one another in the surface biofilm, none of the explanations offered for distinct dentinal tubule localization are entirely satisfactory. Some additional discussion would improve the paper.*

Additional discussion related to possible explanations for why these species have distinct localizations in tubules was added to the Discussion (lines 350-362).

**Technical issues and responses:**

*1) We notice that your supplementary figures are uploaded with the file type 'Figure'. Please amend the file type to 'Supporting Information'. Please ensure that each Supporting Information file has a legend listed in the manuscript after the references list.*

We have made these changes in the online submission and added legends for Supplemental Figure S4 and the three Supplemental Tables in lines 974-992 *2) Please ensure that the CRediT author contributions listed for every co-author are completed accurately and in full. At this stage, "Several Authors" require contributions. Please ensure that the full contributions of each author are acknowledged in the ""Add/Edit/Remove Authors"" section of our submission form.

NOTE: Please update the Authors Contribution in the ONLINE SUBMISSION FORM.*

We have updated the online submission form accordingly. *3) Thank you for stating"Other data is included within"

Please provide a complete Data Availability Statement in the submission form, ensuring you include all necessary access information or a reason for why you are unable to make your data freely accessible.
If your research concerns only data provided within your submission, please write "All data are in the manuscript and/or supporting information files" as your Data Availability Statement.
If your research concerns data from external sources, Please amend your Data Availability Statement to include the full link to the data*

We have amended the data availability statement to include links to SRA and github where sequence data and QPCR data is archived. *4) Please provide an Author Summary. This should appear in your manuscript between the Abstract (if applicable) and the Introduction, and should be 150–200 words long. The aim should be to make your findings accessible to a wide audience that includes both scientists and non-scientists.*

An author summary is now in lines 71-84 of the manuscript

*5) We have noticed that you have cited "Table S1" in the manuscript file. However, there are no corresponding file uploaded to the submission. Please upload it as separate files with the item type 'Supporting Information'.*

We uploaded the table as supporting information. *6) We have noticed that you have uploaded Supporting Information files, but you have not included a list of legends [SI Tables] . Please add a full list of legends for your Supporting Information files after the references list.*

We have included the legends – see above. 

*7) Thank you for stating" The subjects for microbiome analysis were recruited from patients at the Nationwide Children’s Hospital dental clinics using an Institutional Review Board (IRB)-approved protocol (NCH 00000478). They ranged in age from 24-59 months old with a median of 48. The subjects for imaging analysis were recruited at the Department of Dentistry at Boston Children’s Hospital usingan IRB-approved protocol (IRB-P00030469)

Please include the name(s) of the Institutional Review Board(s) or Ethics Committee(s)*

The full names are now given in lines 422-426.
